# Assessment of the Advancement of Alveolar Bone Loss in Canine Patients Receiving Definitive Radiotherapy for Sinonasal Tumors

**DOI:** 10.1111/vru.70173

**Published:** 2026-04-20

**Authors:** Erin Paul‐Stewart, Kekauilani Zukeran‐Kerr, Alex Pinto, Christopher Snyder, Nate Van Asselt

**Affiliations:** ^1^ Department of Surgical Sciences School of Veterinary Medicine University of Wisconsin—Madison Madison Wisconsin USA; ^2^ Department of Biostatistics and Medical Informatics School of Medicine and Public Health University of Wisconsin—Madison Madison Wisconsin USA

**Keywords:** late effects of radiotherapy, nasal carcinoma, periodontal disease

## Abstract

Radiotherapy for the treatment of sinonasal tumors in dogs is generally well tolerated, but the range of long‐term adverse effects remains poorly understood. In people receiving radiation for head and neck tumors, existing dental disease is a predisposing factor for radiation‐induced oral complications that can lead to worsening periodontal disease long‐term. Periodontal disease is widespread amongst the population of senior dogs typically undergoing radiotherapy, and the aim of this retrospective cohort study was to determine if there is evidence of increased alveolar bone loss in these patients. Computed tomography scans from canine patients undergoing definitive intent radiotherapy for sinonasal tumors at a veterinary teaching hospital from 2013 to 2022 were utilized to assess alveolar bone loss in the maxillary and mandibular premolars and molars at multiple follow‐up time points. As the radiation dose delivered was concentrated to the maxilla, the mandibular teeth served as a control for the expected degree of alveolar bone loss. Analysis indicated that there is an increase in alveolar bone loss over time, but that there is no difference between the mandible and maxilla. This finding provides evidence that the risk of radiotherapy worsening alveolar bone loss is low in canine patients and is the first study evaluating the relationship between alveolar bone loss and radiation‐induced side effects in dogs. Continued long‐term follow‐up, along with similar investigations for tumors more directly affecting the oral cavity, is essential to appropriately guide patient risk assessment and prophylaxis.

## 1 Introduction

Radiotherapy is the cornerstone for the treatment of head and neck tumors in both people and dogs. In dogs, some of the most frequently treated head and neck tumors are sinonasal tumors, most commonly carcinomas and sarcomas [1, 2]. Recent technological advancements have improved radiation dose conformation to sinonasal tumors, but normal tissue is still often included in high dose regions, resulting in adverse effects of radiation. Short‐term adverse effects of radiotherapy for sinonasal tumors in dogs include mucositis, dermatitis, alopecia, keratitis, and conjunctivitis [2, 3, 4, 5]. Long‐term adverse effects are rare and less well defined but include leukotrichia, cataracts, and fistulas [6]. Definitive radiotherapy for sinonasal tumors in dogs results in average survival times of 12–18 months [3, 5, 7], and with technological advancements that are increasing survival times, long‐term adverse effects are of increasing importance.

In people receiving radiation for head and neck tumors, preexisting dental disease has been found to worsen radiation‐induced oral complications, including mucositis, periodontal attachment loss, and tooth decay [8, 9]. Bony structures exposed to radiotherapy are often abnormal, and later treatments such as surgically manipulating hard and soft tissues can lead to osteonecrosis of the jaw [10, 11]. The proposed pathophysiology for this relationship involves the acute pro‐inflammatory state associated with mucositis combined with the direct effects of radiotherapy on periodontal tissue: decreased tissue perfusion, tissue fibrosis, and capillary destruction, resulting in an oral environment that promotes progression of periodontal disease [12, 13]. Overall, quality of life is negatively impacted due to worsening in oral health and function, pain, dental caries, and instances of osteonecrosis. To prevent these short and long‐term complications in people, pre‐irradiation dental care, including management of periodontal disease, is crucial for human head and neck patients and widely implemented [14]. The frequency of dental evaluations and cleanings is often increased when a person undergoes radiation treatment to help minimize the risk of osteoradionecrosis.

Despite the strong relationship that has been established between periodontal disease and oral health after radiotherapy in people, little is known about the effect of radiotherapy on oral health in dogs. The prevalence of periodontal disease in dogs is frequent, with approximately 80% of dogs over 3 years of age affected [15]. As periodontal disease progresses, it has been associated with severe local and systemic sequelae that can contribute to decreased quality of life and frequent need for veterinary care [16]. Considering the age of the population of dogs most commonly undergoing radiotherapy for head and neck tumors, understanding the effects on oral health is essential to guide veterinarians and owners’ awareness of potential late side effects. There is no known published literature evaluating the relationship between periodontal disease stage and radiation‐induced side effects in dogs. In addition, knowledge about late side effects following radiation is generally limited due to loss of veterinary patients to follow up, which is typically when these side effects are observed in people. In one study of dogs undergoing radiotherapy for nasal tumors, a total of three dogs were found to have developed dental caries and dental pulp death in the radiation field several years after treatment [17]. Another study discussed 13 cases of osteonecrosis of the jaw in dogs that occurred within previously irradiated fields [18]. Several of these cases developed osteonecrosis following dental extractions, which is consistent with one of the most frequently reported inciting factors in people.

Considering these limited reports, further information is necessary to determine if pre‐irradiation dental care in dogs would be beneficial. The purpose and aim of this study were to further evaluate the impact of radiotherapy on alveolar bone loss in dogs by determining if alveolar bone loss advances more rapidly in dogs undergoing definitive radiotherapy for sinonasal tumors. It was hypothesized that radiotherapy does not cause more rapid advancement of alveolar bone loss in these patients. Information gathered from this study will be used to guide continued investigations into the impact of radiotherapy on periodontal health and make recommendations to ensure improvement in the quality of life for patients and decrease morbidity associated with radiation.

## 2 Materials and Methods

### 2.1 Patient Selection

This study was a retrospective, cohort design. Due to the retrospective nature of the study, no ethical approval was required. Record review and patient selection were performed by a small animal rotating intern veterinarian under the supervision of a board‐certified radiation oncologist. Records for canine patients with spontaneous primary sinonasal tumors undergoing a definitive‐intent radiation protocol (4.2 Gy/fraction × 10 daily fractions) at University of Wisconsin–Madison Veterinary Care (UWVC) from 2013 to 2022 were obtained from the radiation therapy (RT) database. The diagnostic imaging database was searched to determine which of these dogs had at least one follow‐up computed tomography (CT) scan performed more than 3 months following the initial RT planning CT scan. These dogs were included in the study. A power analysis was not performed prior to patient selection. Basic demographic data were collected, including age at diagnosis, sex, reproductive status, breed, weight, type of tumor, and survival time.

For the control group, records for canine patients receiving a CT scan of the head at UWVC from 2008 to 2023 were obtained from the medical records database. Records were reviewed to determine which dogs had at least one follow‐up CT scan of the head performed more than 3 months following the initial CT. Of this group, dogs were excluded if their diagnosis and reason for CT of the head would potentially contribute to more rapid or severe progression of alveolar bone loss. The most common reasons for exclusion were receiving RT for a tumor that would result in the oral cavity being exposed to radiation (e.g., nasal and oral) and conditions directly affecting the integrity of the oral cavity (e.g., maxillary or mandibular fractures and tooth root abscesses). The remaining dogs were included as the control group, and demographic data were collected (age at diagnosis, sex, reproductive status, breed, weight, and diagnosis).

### 2.2 Radiation Planning and Treatment

Intensity‐modulated RT was delivered using a 6 MV helical tomotherapy unit (TomoTherapy HiArt or Radixact Treatment System, Accuray Inc., Sunnyvale, CA, USA) and planned with inverse planning software (TomoTherapy HiArt v3‐5, Precision 3.1.0.0) using the convolution‐superposition algorithm. For the pretreatment CT scan, all dogs were positioned in sternal recumbency in a deflatable vacuum cushion. The head was immobilized using a maxillary dental mold bite block. Structures were contoured on precontrast CT image sets and registered to postcontrast images. Slice thickness ranged from 1.25 to 2.5 mm. Gross tumor volume (GTV) included contrast‐enhancing mass in all patients. A 2 cm clinical target volume (CTV) expansion was made cranially and caudally from the GTV within the affected nasal cavity and sinuses, including any non‐enhancing fluid, respecting the anatomic borders of tumor extension. In dogs with subcutaneous tumor extension, CTV margins in the subcutaneous tissues were variable. A 2 mm isotropic planned tumor volume (PTV) was created to ensure irradiation of the CTV and was bound by the body surface.

All dogs were prescribed 10 fractions of 4.2 Gy to the PTV. The treatment planning optimization aims for target coverage at our institution, which is to deliver at least 95% of the prescribed dose to at least 95% of the PTV with a maximum dose of ≤107%. A dose volume constraint of 60% of the eyes to receive <15 Gy (*D*60% < 15 Gy) was attempted in all patients 7. Dosimetry for each case was verified by a medical physicist using either an ion chamber for point dose measurement and film for planar comparison with the calculated dose or a biplanar diode array dosimeter (Scandidos Delta4 System, Scandidos, Sweden). Patient positioning was verified with on‐board megavoltage or kilovoltage CT prior to each fraction.

### 2.3 Data Collection

All CT scans performed posttreatment for each dog were categorized as 3–6, 6–9, 9–12, or greater than 12 months post‐RT treatment. These time frames were based on standard recommendations for post‐RT follow‐up. For the control group, the same categories were used for CTs performed following the initial CT at diagnosis. If dogs had multiple CT scans performed within a 3‐month window, the later CT scan was selected for analysis. The dates of all follow‐up CT scans were cross‐referenced with dates of a second course of RT, if performed. One dog had a CT scan in the >12‐month window that was performed after a second, palliative (4 Gy × 5 fractions) RT treatment. This data were included in the analysis.

**FIGURE 1:**
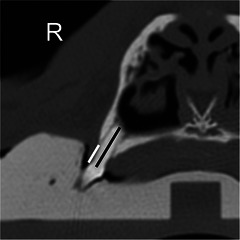
Alveolar bone loss was assessed on CT images by measuring the distance from the alveolar crest to the cementoenamel junction (white line). This value was divided by the distance from the tooth root apex to the cementoenamel junction (black line) to determine the percentage of alveolar bone loss. This percentage was converted to an overall grade per tooth (Grades 1–3 as described previously) based on the greatest percentage of alveolar bone loss for any root of that tooth.

CT scans were acquired using a GE Discovery RT Gen 2 (GE Healthcare, Chicago, IL, USA) in the bone window with technique settings of 120 kVp and 175–250 mA s. CT data collection was performed by a second‐year resident in veterinary diagnostic imaging using a free, open‐source medical imaging viewer (Horos, v4.0.0). The radiology resident worked with a boarded radiologist to ensure a standardized approach to the evaluation of the CT images. The resident was not blinded to the status of the dog or the timing of the CT scan relative to RT. Each tooth was evaluated individually using 3D multiplanar reformats to optimize the evaluation of surrounding alveolar bone loss. A grading system for classifying the degree of alveolar bone loss on CT scan was created for the purpose of this study. Grades were defined based on the percentage of alveolar bone loss. Alveolar bone loss was assessed by the method described in Figure 1, and grades were assigned as follows: Grade 1 (<25% loss); Grade 2 (25%–50% loss); Grade 3 (>50% loss). All maxillary and mandibular premolar and molar teeth were assigned a grade according to the defined system based on percent alveolar bone loss. Any missing teeth were assigned a grade of “0” for data collection purposes and were not included in statistical analysis.

Using the RT planning software, the planning CT for each patient was utilized to determine the approximate radiation dose delivered to all premolar and molar teeth. The approximate center of each tooth was located on CT, and the reported dose delivered in that location was recorded. All dogs except one had accessible RT plans for dose data collection.

### 2.4 Statistical Analysis

Basic descriptive statistics (minimum, maximum, and mean) were calculated for GTV and PTV data to assess RT plans. Median survival time was calculated using Kaplan–Meier analysis, with survival time being defined as the time from RT planning CT scan to date of death. The generalized estimating equations (GEE) model with alternating logistic regression (ALR) was used to analyze the grade of alveolar bone loss over time. The ALR approach accounts for correlation attributable to repeated measures on teeth within dogs. To analyze the change in grade of alveolar bone loss over time, time was modeled continuously (unit = 1 month) to account for arbitrary time categories for follow‐up CTs and the wide range of follow‐up time long term.

Model findings were presented as odds of an increase in grade of alveolar bone loss over time for all teeth compared to baseline and for maxillary compared to mandibular teeth. This same analysis was performed for the smaller subset of dogs (*n* = 6) that had a CT scan performed at each of the recommended intervals (a total of four CT scans in addition to RT planning CT). Analysis of the mandibular teeth was also performed in the control group. A GEE model was used to assess whether the mandible could be used as an intra‐patient control by comparing the change in grade of alveolar bone loss over time of the mandible in patients receiving radiation versus the control group of patients that did not receive radiation. The Wilcoxon rank sum test was used to compare the median age and weight of patients in the experimental versus the control group. A *p* value of less than 0.05 was considered statistically significant. All statistical analyses were performed using SAS software version 9.4 (SAS Institute, Cary, NC, USA).

## 3 Results

### 3.1 Patient Characteristics—Experimental Group

A total of 32 dogs met the inclusion criteria of undergoing definitive‐intent (4.2 Gy × 10 fractions) radiotherapy for a spontaneous sinonasal tumor at UWVC between 2013 and 2022 and having at least one CT scan performed more than 3 months following the initial RT planning CT. The patient population consisted of 21 male dogs and 11 female dogs, all neutered. Breeds represented included mixed breed (*n* = 6), Labrador Retriever (3), Border Collie (3), Australian Cattle Dog (2), Golden Retriever (2), Pembroke Welsh Corgi (2), and Jack Russell Terrier (2); Australian Shepherd, Bichon Frise, Boston Terrier, Bouvier des Flandres, Boxer, Brussels Griffon, Canaan Dog, German Shorthaired Pointer, Pug, Siberian Husky, Wire Fox Terrier, and Yorkshire Terrier were all represented once. The median weight was 22.0 kg (interquartile range 10.7–25.3). The median age at diagnosis was 10.1 years (IQR 7.9–11.4). Histologic category of the sinonasal tumor was available for 20/32 patients and included carcinoma (*n* = 17) and sarcoma (3). Kaplan–Meier analysis was performed to determine median survival time for all dogs for which a date of death was known (*n* = 20). The median survival time for dogs was 17.5 months (10.2, 22.1).

All dogs had an initial CT scan for RT planning. Twenty‐three out of 32 (72%) of dogs had a CT scan available for analysis in the 3–6‐month post‐RT window, 20/32 (63%) in the 6–9‐month window, 10/32 (31%) in the 9–12‐month window, and 10/32 (31%) in the >12‐month window. Six out of 32 dogs (18%) had a CT scan in each of the windows.

### 3.2 Patient Characteristics—Control Group

A total of 27 dogs met the inclusion criteria for the control group. Of these dogs, a total of 15 were excluded due to inadequate slice thickness of CTs performed prior to 2015. The patient population consisted of nine male dogs and six female dogs, all neutered except for one intact male. Breeds represented included mixed breed (*n* = 4), Cocker Spaniel (2); Boston Terrier, French Bulldog, German Shepherd, Golden Retriever, Great Dane, Labrador Retriever, Shih Tzu, Weimaraner, and Yorkshire Terrier were all represented once. The median weight was 20.0 kg (interquartile range 7.3–31.6). The median age at diagnosis was 9.1 years (IQR 4.5–11.0). Diagnoses included brain tumors (*n* = 4), nasal aspergillosis (3), periorbital swelling (2), otitis media/interna, rhinitis, cervical intervertebral disc herniation, salivary adenocarcinoma, sialocele, and mandibular abscess.

All dogs had an initial CT scan at diagnosis. Nine out of 15 (60%) of dogs had a CT scan available for analysis in the 3–6‐month post‐diagnosis window, 4/15 (27%) in the 6–9‐month window, 3/15 (20%) in the 9–12‐month window, and 6/15 (40%) in the >12‐month window. One out of 15 dogs had a CT scan in each of the windows.

### 3.3 Radiation Planning and Dose

The mean dose of radiation delivered to maxillary teeth (95% confidence interval) was 29.4 Gy (27.7, 31.0), whereas the mean dose delivered to mandibular teeth was 12.5 Gy (11.8, 13.2). This was a significant difference (*p* < 0.01). As shown in Table 1, the average dose of radiation delivered to the tumor was appropriate.

**TABLE 1:** Descriptive statistics for dose of radiation delivered to the tumor for included patients (*n* = 31).

	Minimum (Gy)	Maximum (Gy)	Mean (Gy)
GTV 98%	39.8	42.2	41.7
GTV 50%	42.3	44.2	42.8
GTV 2%	42.6	46.1	43.5
PTVeval 98%	36.1	41.8	40.5
PTVeval 50%	42.2	44.1	42.7
PTVeval 2%	42.8	45.7	43.6

Abbreviations: GTV, gross tumor volume; PTV, planned tumor volume.

### 3.4 Intra‐Patient Mandibular Control

Prior to assessing alveolar bone loss in the maxilla versus mandible of patients receiving radiation, the control group was used to determine if the mandible could be used as an intra‐patient control for expected alveolar bone loss over time, as it receives significantly less radiation in patients with sinonasal tumors. It was hypothesized that the change in grade of alveolar bone loss in the mandible of patients receiving radiation would not differ from that of the mandible of the control patients who did not receive radiation. On the basis of the GEE model estimates, there was no difference in the change in grade of alveolar bone loss over time between these two groups (*p* = 0.7614).

There was no difference between the age at diagnosis and weight of the experimental versus control groups (*p* = 0.0769 and 0.8106, respectively).

### 3.5 Alveolar Bone Loss

For all dogs, a total of 762 different teeth were assigned grades over time for a total of 2276 grades. For the six dogs with follow‐up CT scans in all recommended time frames, a total of 146 teeth were assigned grades over time for a total of 730 grades.

## 4 Discussion

**FIGURE 2:**
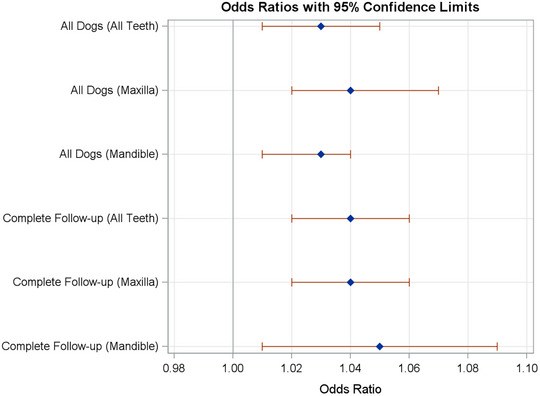
Odds of a tooth having a higher grade of alveolar bone loss for each increase of 1 month from baseline. Odds ratios represent the change in likelihood of increased bone loss over time. Complete follow‐up includes dogs with follow‐up CTs in all post‐RT windows as described in the methods.

In this study, serial CT scans from canine patients with sinonasal tumors were analyzed to assess the progression of alveolar bone loss following definitive radiotherapy. The population of dogs, treated at UWVC from 2013 to 2022, consisted mostly of older, large breeds with carcinomas and sarcomas, which was representative of the typical signalment of dogs treated for sinonasal tumors [2, 3]. All patients completed adequate definitive‐intent (4.2 Gy × 10) radiotherapy protocols (Table 1). Maxillary and mandibular premolar and molar teeth were assigned a grade of alveolar bone loss on CT scans using a system created for this study (Figure 1). A continuous statistical model was used to assess for an increase in the grade of alveolar bone loss over time. This analysis showed there is a small increase in the odds of a tooth being assigned a higher grade over time (Table 2, Figure 2), which is expected considering that periodontal disease and associated bone loss are a progressive condition [15]. As the maxillary teeth receive a significantly higher therapeutic dose of radiation compared to the mandibular teeth, the mandibular teeth served as a control group to assess whether the dose of radiation is associated with increased alveolar bone loss. The use of the mandible as an intra‐patient control was validated by analysis confirming no difference in the change in grade of alveolar bone loss in the mandible of patients receiving radiation for a nasal tumor versus control patients that did not receive radiation. There was no statistically significant difference between the odds of a tooth being assigned a higher grade if it was located on the maxilla versus the mandible in patients receiving radiation (Table 2). In addition, the difference in odds ratios was minimal. This supports the conclusion that radiation delivered with a definitive‐intent protocol for sinonasal tumors does not accelerate worsening alveolar bone loss within the median survival time for these patients.

**TABLE 2:** Odds of a tooth having a higher grade of alveolar bone loss for each increase of 1 month from baseline.

	All dogs (*n* = 32) Odds ratio (95% CI)	Dogs with follow up CTs in all post‐RT windows (*n* = 6) Odds ratio (95% CI)
All teeth	1.03 (1.01, 1.05)^*^	1.04 (1.02, 1.06)^*^
Maxilla	1.04 (1.02, 1.07)	1.04 (1.02, 1.06)
Mandible	1.03 (1.01, 1.04)	1.05 (1.01, 1.09)

*Note*: For all teeth compared to baseline, *p* < 0.01 (*) for both sets of dogs. For the maxilla compared to the mandible, *p* = 0.18 and 0.65, respectively.

Due to the variety in times post‐RT that the CT scans were performed for most dogs, statistical analysis was repeated in the smaller group of dogs that had a CT scan performed at each of the recommended follow‐up intervals, and therefore, the longest degree of follow‐up. The same results were obtained (Table 2, Figure 2), which further supports the lack of contribution of radiation to the progression of alveolar bone loss during the time frame of this study. There was one dog who received a second, palliative course of RT prior to the >12‐month CT scan. Although this data were not excluded from analysis, if it is assumed that a second course of RT would have predisposed this patient to worsening periodontal disease and therefore skewed the results in this direction, including this patient did not appear to significantly affect the analysis.

Several limitations of the data collected in this study may have played a role in the interpretation of results. As mentioned previously, several dogs had multiple CT scans performed within one time category, and the later one was selected for analysis. Although these means some data on alveolar bone loss were not included in the analysis, any additional data would have been from earlier time points and therefore would have further supported the lack of a difference in alveolar bone loss between the mandible and maxilla. An additional limitation is that teeth were categorized based on alveolar bone loss instead of measuring the exact percentage at each time point, and therefore some teeth may have had progressive bone loss that was not to a degree that would result in a jump in category but may have been statistically significant if measured and analyzed for each patient. Very few teeth received an initial grade of 3, and therefore, the impact of this being the maximum grade is likely minimal. Obtaining these measurements and associated analysis would be beneficial in future studies. In addition, future studies should attempt to include a larger group of patients, as the lack of a significant difference in progression between groups could be related to Type II error.

Another variable is that dogs were not included or classified based on the stage of their tumor, and therefore, including all stages in the same analysis may have masked true worsening of periodontal disease in patients with more invasive tumors. Eight dogs had alveolar bone loss associated with bony lysis from the tumor; however, all this bone loss was noted on initial planning CT scans and therefore is unlikely to have affected analysis. Another potential confounding variable is that most dogs included in this study were considered medium to large breeds, which are typically at a lower risk for periodontal disease [16, 19]. It is possible that smaller breed dogs or those with more severe existing periodontal disease may be more prone to radiation‐induced adverse effects on the oral cavity; however, this difference would be more important to consider on an individual patient basis, as most dogs with sinonasal tumors are medium to large breeds. Finally, the data analyzed in this study spanned approximately 18 months following RT treatment, with the majority of data concentrated prior to 12 months. Although this time span aligned with the median survival time of dogs in this study, there are many dogs with a longer survival time. Considering the wide range of time (months to 5 years) when progressive periodontal disease or radiologic effects such as osteoradionecrosis occur in people and dogs [10, 18], assessment of alveolar bone loss on CT scans further out from treatment would provide a more complete assessment of long‐term risk.

Although this study showed that radiation is not associated with progression of alveolar bone loss, this was the only objective sign of periodontal disease analyzed. To obtain a complete picture of oral health in dogs undergoing radiotherapy, other criteria such as oral exam findings throughout treatment, additional measures of periodontal and endodontal disease on CT, incidence of mucositis, and changes in quality of life should be included in any prospective study design. Radiotherapy has been shown to affect the oral microbiome in people, and this may represent an emerging avenue for investigation [20]. A prospective study should also include collecting data on historic extractions or other dental procedures and standardizing whether patients receive other treatments such as anti‐inflammatory medications, chemotherapy, or nasal cavity exenteration during the study period. The format for this retrospective study can also be applied to other tumor types where radiotherapy may have an impact on the oral cavity, namely, oral tumors such as squamous cell carcinoma, melanoma, and fibrosarcoma.

On the basis of the results of this study, radiotherapy does not contribute to measurable progression of alveolar bone loss in canine patients with sinonasal tumors. However, as survival times continue to improve with the implementation of new radiotherapy technologies and as protocols such as stereotactic radiotherapy become more popular, the impact of radiotherapy on periodontal health should continue to be reexamined. The clinical effects of radiotherapy on periodontal health in canine patients are not fully elucidated, and the risk for similar adverse effects as seen in people is still present. Performing an oral exam and recommending intervention for existing periodontal disease that is severe or affecting quality of life should continue to be a part of the workup for all patients receiving radiotherapy for sinonasal tumors. Individual patient characteristics and the recognition that delaying radiotherapy may affect tumor progression should always be factored in, and the ideal timing of whether to address periodontal disease before or after radiotherapy is unknown. This study did show that progressive alveolar bone loss does occur over time in canine patients, and therefore recommending at‐home dental care based on guidelines from established organizations (AVDC, AAHA, and Veterinary Oral Health Council) can only benefit radiotherapy patients prior to, during, and following treatment. Further studies are necessary to determine if pre‐irradiation dental care is indicated for at‐risk breeds, dogs with a history of severe periodontal disease, or dogs with increased destruction of alveolar bone due to tumor burden. Identifying these at‐risk dogs is essential for appropriately counseling clients, decreasing morbidity associated with radiotherapy, and improving the quality of life in veterinary radiotherapy patients.

## Author Contributions

**Erin Paul‐Stewart**: investigation, writing – original draft, writing – review and editing, and data curation. **Christopher Snyder**: conceptualization, writing – review and editing, methodology, and resources. **Nate Van Asselt**: conceptualization, investigation, writing – original draft, writing – review and editing, methodology, project administration, supervision, and resources. **Kekauilani Zukeran‐Kerr**: conceptualization, investigation, writing – original draft, methodology, writing – review and editing, formal analysis, and data curation. **Alex Pinto**: conceptualization, writing – original draft, methodology, writing – review and editing, and formal analysis.

## Disclosure

The EQUATOR network STROBE guidelines for reporting observational studies were used for this study.

## Conflicts of Interest

The authors declare no conflicts of interest.

## Data Availability

To access data supporting the results in this article, please contact the corresponding author.
